# Current Distribution of the Olive Psyllid, *Euphyllura olivina*, in California and Initial Evaluation of the Mediterranean Parasitoid *Psyllaephagus euphyllurae* as a Biological Control Candidate

**DOI:** 10.3390/insects11030146

**Published:** 2020-02-26

**Authors:** Evelyne Hougardy, Xingeng Wang, Brian N. Hogg, Marshall W. Johnson, Kent M. Daane, Charles H. Pickett

**Affiliations:** 1 Invasive Species and Pollinator Health Research Unit, USDA-ARS, Albany, CA 94710, USA; brian.hogg@usda.gov; 2 Department of Environmental Science, Policy and Management, University of California, Berkeley, CA 94720, USA; Xingeng.Wang@usda.gov (X.W.); kdaane@ucanr.edu (K.M.D.); 3 Beneficial Insects Introduction Research Unit, USDA-ARS, Newark, DE 19713, USA; 4 Department of Entomology, University of California, Riverside, CA 92521, USA; mwjohnson@ucanr.edu; 5 California Department of Food and Agriculture, Biological Control Program, Sacramento, CA 95832, USA; charlie.pickett@cdfa.ca.gov

**Keywords:** olive psyllid, *Euphyllura olivina*, *Psyllaephagus euphyllurae*, California, distribution, foreign collections, classical biological control, non-target assessment

## Abstract

The olive psyllid, *Euphyllura olivina*, is a newly invasive species to California with the potential to become an economical pest if it reaches the olive production regions of California’s Central Valley. Here, we report on surveys undertaken in California to assess the psyllid’s current distribution and the occurrence of parasitism. Additionally, we present results of foreign collections of its parasitoids and initial non-target studies of a possible biological control agent, the Mediterranean parasitoid *Psyllaephagus euphyllurae*. The current distribution of the psyllid appears to be limited to the California coast between Monterey and San Diego; there have been no reports of infestations on olives in the major production areas of central and northern California. *Psyllaephagus euphyllurae* was the major primary parasitoid found in our foreign collections. The potential non-target impact of *P. euphyllurae* was tested on three native North American psyllid species: *Neophyllura* *arctostaphyli*, *Euglyptoneura* nr. *robusta*, and *Calophya* *nigrella*. No *P. euphyllurae* developed on the non-target species during no-choice tests. Behavioral observations in choice tests confirmed no attack on the non-target hosts, although the parasitoid did remain longer on *N. arctostaphyli*-infested manzanita plants, and revealed no host feeding behavior.

## 1. Introduction

The olive psyllid, *Euphyllura olivina* (Costa) (Hemiptera: Liviidae), native to southern Europe, was first reported on olives (*Olea europaea* L.) in Orange County, California (USA), in 2007 [1]. *Euphyllura olivina* is an economic olive pest in its native region, feeding almost exclusively on the flower blossoms and soft growing tissue of olive. It is mostly abundant in spring when olive trees are flowering and causes up to 60% yield loss in some parts of the Mediterranean Basin [2,3]. 

*Euphyllura olivina* is not currently considered an economic pest in California because it has not yet reached the olive production region of California’s Central Valley, which has a very similar climate to southeastern Spain where the psyllid is widely established. The USDA APHIS New Pest Advisory Group (NPAG—report 20071218, 2007) classified the olive psyllid as a reportable non-actionable pest and recommended a classical biological control approach rather than a phytosanitary strategy. Classical biological control (i.e., introduction of co-evolved natural enemies) is a valuable option for permanently suppressing populations of established and incipient pests in commercial orchards and in ornamental landscapes, where more intensive management methods such as chemical control may be prohibitively expensive and/or environmentally undesirable. Thus, the introduction and permanent establishment of a parasitoid specializing on the olive psyllid in California would provide an efficient means of controlling and slowing the spread of this pest.

*Psyllaephagus euphyllurae* (Masi) (Hymenoptera: Encyrtidae) is the most common primary parasitoid associated with this psyllid in its native range [4,5,6]. The genus *Psyllaephagus* Ashmead consists of more than 200 species, most being primary endoparasitoids specific to species of Psyllidae [7]. Representatives from this genus have been successfully used in classical biological control projects targeting various pest species of Psylloidea. Recent examples include *P. pilosus* Noyes for control of eucalyptus psyllids in Ireland [8] and *P. bliteus* Riek for control of red gum psyllids in California [9,10].

*Psyllaephagus euphyllurae* is a thelytokous (female only population that reproduces by parthenogenesis) solitary endoparasitoid with a preference for late psyllid instars [11]. No data on adult longevity nor fecundity are available in the literature. In the western region of the Mediterranean basin, the parasitoid appears to be bivoltine, entering a resting state as a pre-adult in a mummified host (melanized exoskeleton) in early summer [12]. This resting state is synchronized with the lack of hosts during the summer months when the olive psyllid enters a reproductive diapause [2,13]. Both the psyllid and *P. euphyllurae* do not resume activity until mid-winter to early spring the following year. This host–parasitoid synchronicity suggests that *P. euphyllurae* is highly specific to *E. olivina*.

This study reports on (1) Californian surveys undertaken since 2009 to assess the current distribution of the olive psyllid and any local parasitoids, (2) foreign collections of parasitoids associated with the olive psyllid in its region of origin, and (3) a limited assessment of the parasitoid’s potential non-target impact and host range using three native North American psyllid species. 

## 2. Materials and Methods 

### 2.1. Survey of the Olive Psyllid and Local Parasitoids in California

Surveys were conducted during spring months of 2009, 2010, 2011, 2014, 2015, and 2018 when olive psyllid nymph populations would have been highest. Initial surveys in 2009 and 2010 were conducted in San Diego and Orange counties of southern California. Afterwards, surveys were extended north to San Francisco. All surveys were conducted on ornamental trees free of pesticides, some of which were at Spanish missions established in the late 1700s to early 1800s along the California coast. The presence of psyllids was assessed by spending 5–10 min searching the canopy and suckers for the waxy excretions produced by the insects. The number of trees sampled at any one site varied from 1 to 50, proportionally to the number of trees present. The numbers of collection sites each year varied from 27 to 68.

Local parasitoids that could be attacking this psyllid were also surveyed. Sampling of psyllid parasitoids was conducted in 2009, 2014, 2015, and consisted of collecting cuttings from the surveyed sites. The first collection in 2009 was conducted in San Diego County, the first area in the state known to have olive psyllid populations. Ten trees were sampled for wax infested stems harboring psyllid nymphs on 10 June 2009: five from the Presidio public park in San Diego, and five found along public roads in Carlsbad. Cuttings were placed in cages constructed of plastic Ziploc bags, modified with a screen opening to allow for ventilation. Bouquets of two or three 0.2 m length stem cuttings were placed into water-saturated floral foam^®^, which in turn was wrapped in parafilm. The cages were held either at the University of California Riverside Quarantine Facility or at the CDFA Quarantine facility in Sacramento. Two months later, cages were examined for the presence of parasitoid adults and mummies. For surveys in 2014 and 2015, stems with nymphs were placed into large bags and exposed to ambient conditions for 2 to 4 weeks to allow for development of potential parasitoids. 

In addition to these surveys, the Pest Detection Reports (PDR) database was examined for the frequency of olive psyllid identifications during the first 12 years of the pest’s presence in California. These reports provide an indirect measure as to the change in this psyllid’s population size and distribution. They represent arbitrary submissions of insects collected by anyone in California to CDFA’s Plant Pest Diagnostics Center for identification to species. 

### 2.2. Foreign Collection of Olive Psyllid Parasitoids

Collections of parasitized (mummified) olive psyllids were conducted in 2013, 2014, 2015, and 2018 in Spain, in the coastal regions of Catalonia, Valencia, and southeast and west of Murcia (Table 1) on psyllid-infested olive trees in abandoned and active commercial organic orchards throughout each area. Field-collected mummies were placed in 0.5 dram glass vials plugged with cotton and were shipped under the appropriate permit to the University of California Riverside Quarantine facility (2013, 2014, and 2015) or the University of California Berkeley Quarantine facility (2018) where they were monitored for adult emergence. Parasitoids were preserved in 95% ethanol to confirm their identification. Voucher specimens for parasitoids collected in Spain for the years 2013, 2014 are deposited at the Entomology Research Museum, University of California, Riverside. Those collected in 2018 have voucher specimens deposited at the same university museum, in addition to the United States National Museum in Washington D.C., the Essig Museum at the University of California Berkeley, and the California State Collection of Arthropods in Sacramento.

### 2.3. Non-Target Impact of *P. euphyllurae*

Three species of native psyllids were selected based on their relatedness to the olive psyllid, their occurrence in habitats near commercial olive orchards, and/or their availability. *Neophyllura arctostaphyli* Schwarz (Hemiptera: Liviidae) is native to California and is a close relative to the target species (both in the subfamily Euphyllurinae). It is usually found on *Arctostaphylos* spp. (manzanita). The two other psyllids selected are associated with native plants common to the regions of central and northern California: *Euglyptoneura* nr. *robusta* (Crawford) (Hemiptera: Psyllidae) found on *Ceanothus* spp. and *Calophya nigrella* Jensen (Hemiptera: Calophyidae) found on *Rhus trilobata* Nutt (skunkbush sumac).

Non-target species for the host specificity tests came directly from field collections in California. Infested manzanita cuttings were collected in El Dorado and Napa counties. Infested deerbrush (*Ceanothus integerrimus* Hook. & Arn.) cuttings were collected in El Dorado County, and skunkbush sumac cuttings were collected in Siskiyou County. The target species, *E. olivina*, was also field collected from infested olive trees near Carmel in Monterey County. All Psylloidea vouchers are deposited at the California State Collection of Arthropods in Sacramento. 

Culturing of the parasitoid *P. euphyllurae* is challenging because both the parasitoid and the host psyllid appear to enter obligate resting states during the summer months. Thus, *P. euphyllurae* wasps used in this study originated from mummified psyllids collected in 2018 on psyllid-infested olive trees in Spain. The resulting adult *P. euphyllurae* wasps, all origins combined, were placed in glass vials and provided with honey until they were used in the host specificity tests. All other parasitoids were preserved in 95% ethanol to confirm their identification.

Two sets of experiments were conducted to assess *P. euphyllurae* host specificity. In sequential no-choice tests, *P. euphyllurae* females were presented with the target and non-target species sequentially, and parasitism rates were recorded. In choice tests, *P. euphyllurae* females were presented simultaneously with a choice of target and non-target species and their behavioral responses were recorded. All experiments and observations were conducted in the UC Berkeley Quarantine facilities at 23 ± 2 °C, 16:8 L:D, 50% RH. 

#### 2.3.1. No-Choice Tests

Psyllid-infested plant cuttings were presented to the parasitoids in small bouquets enclosed in ventilated cages (14 × 10.5 cm diam.). To prevent desiccation of the cuttings, the stems were wrapped with cotton and tightly fitted through the lid of a small container filled with water. A honey/water solution was spread on the wall of the cages as a food source for the parasitoids. Each replicate consisted of one naïve, 1–5 d old *P. euphyllurae* female released into a cage containing either the non-target or target species. After 24 h, females were transferred to new cages containing the other host type (if exposed to the non-target species first, they were transferred to a cage containing the target host, and reciprocally). To account for the limited availability of olive psyllid-infested cuttings at the time of parasitoid emergence, females were released in groups of three or five in target cages while always released singly in non-target cages. After the end of the second 24-h exposure, wasps were removed, and the psyllids were left to incubate for 2–3 weeks after which cages and plant materials were examined to determine the number of parasitized nymphs (mummies) and the presence of psyllids and adult wasps. 

#### 2.3.2. Choice Tests

Each replicate consisted of one naïve, 1–12 d old *P. euphyllurae* female (a wide range of ages were used because of limited parasitoid availability) released into a small petri dish (50 mm diameter) containing two small leaves or plant parts; one infested with the target host and one infested with the non-target species. Leaves or other plant parts were sometimes cut into smaller pieces to ensure they were of similar sizes between the two choices and across replicates. Efforts were made to have 2–3 nymphs of mixed ages on each leaf/plant part. Host plant materials were placed parallelly 2 cm apart and the wasp was released at an equal distance from them. Parasitoid behavior was then observed and recorded using a Leica 4EZW microscope and the Leica Acquire software. 

Preliminary observations were conducted to define the following distinctive behaviors: (1) resting, i.e., sitting motionless with the antennae stretched out; (2) grooming, i.e., repeatedly brushing ovipositor or wings with hindlegs, rubbing legs together, or any other actions taken to clean body parts; (3) walking, i.e., moving along the substrate at a relatively constant speed with the antennae stretched out; (4) antennating, i.e., palpating the substrate with the antennae held close together (5) probing, i.e., quickly inserting ovipositor back and forth into the substrate; and (6) ovipositing, i.e., sitting motionless with ovipositor inserted into the host. An additional behavior was included but was never observed: (7) host feeding, i.e., feeding on hemolymph oozing from a wound inflicted with the ovipositor, either by puncturing or ripping open the host cuticle. These observations were scored by two individuals properly trained for objectivity.

After release, females that did not display an obvious searching behavior (i.e., walking and antennating) within 20 min were discarded. Parasitoid behavior was continually observed until either a psyllid was attacked, or the parasitoid left and rested outside of the host patch (i.e., the leaf or plant part) for at least 2 min. The presence of psyllids was confirmed at the end of each observation by lifting the wax covers. Observations were repeated until there were 10–15 replicates for each non-target species. Behavioral data were summarized for each replicate as follows: (1) first choice (=first host plant species encountered), (2) host patch time (=time spent on host plant), (3) occurrence of probing, host finding, attacks (when the parasitoid attempted to parasitize a psyllid), and oviposition.

As advanced maternal age, host deprivation, and egg depletion are known factors prompting host feeding behavior in several parasitoid species [14], the occurrence of host feeding behavior was further investigated by conducting a small number of observations with older wasps. Four 16–24 d old females, with previous oviposition experience on the target hosts but kept separately in a glass vial with just water/honey for a week, were tested as described above. 

### 2.4. Data Analysis

Mean host patch time on target (olive) vs. non-target host plants, and mean age of responsive vs. non-responsive females were compared using *t*-tests performed using JMP Pro 14.0 (SAS Institute Inc. Cary, NC, USA). 

## 3. Results

### 3.1. Survey of Olive Psyllid and Local Parasitoids in California

**Figure 1:**
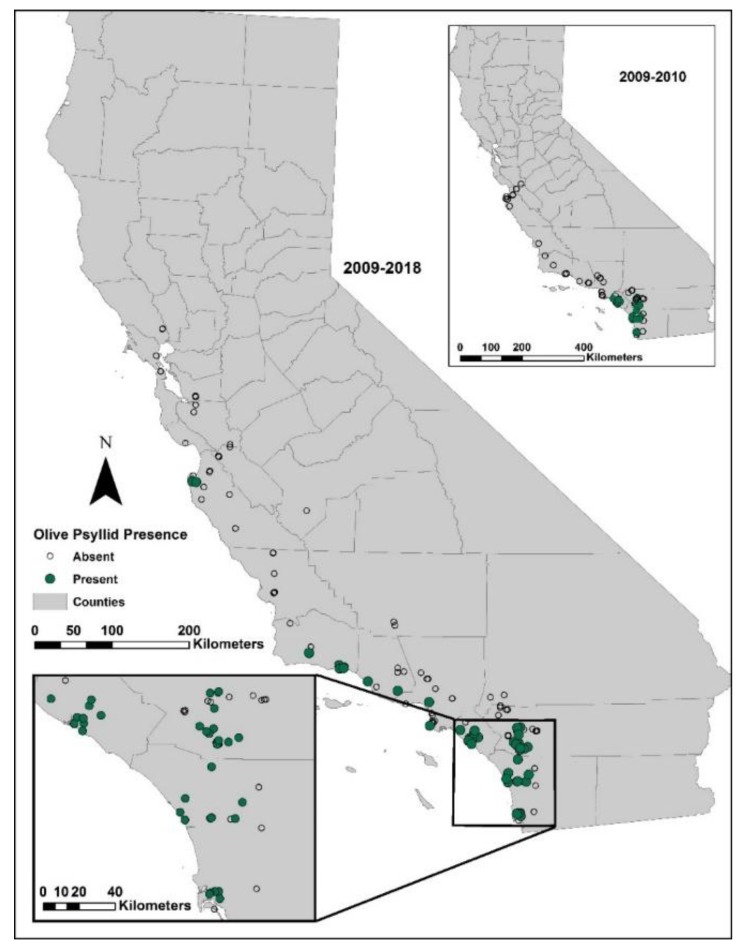
The spread of *Euphyllura olivina* in California based on field surveys conducted in 2009–2018 and analysis of the CDFA Pest Detection Record database.

The change in distribution of the olive psyllid is graphically portrayed by the increase in survey sites found with this pest between 2009 and 2018 (Figure 1). The open and closed circles show that the sampling effort was limited to the coastal regions for central and northern CA, and coastal and inland regions for southern CA, where a greater number of olive trees were found infested by this pest. During the initial year of surveys in southern California (Orange and San Diego counties) 25 of 50 locations had olive psyllid infested trees, and in 2010, 26 of 57 were infested. 

Caged olive cuttings from San Diego County sampled for the presence of parasitoids averaged 3.7 adult olive psyllids (range 1–10), and no parasitoid mummies or adults. No parasitoid developed or emerged from any of the 1058 nymphs collected during the 2014 and 2015 surveys in the California coastal region. Frequency of PDR reports for this pest has increased from one record per year in 2013–2015 to 4–5 records per year in 2017–2019. 

### 3.2. Foreign Collection of Olive Psyllid Parasitoids

The dominant primary parasitoid recovered in the foreign collections was *P. euphyllurae* (Table 2). Two hyperparasitoids, *Apocharips trapezoidea* (Hartig) (Hymenoptera: Figitidae) and *Pachyneuron* sp. (Hymenoptera: Pteromalidae), were the next most abundant species (Table 2). In 2014 and 2015, we also recovered a low proportion of *Psyllaephagus pulchellus* (Mercet), another primary parasitoid.

### 3.3. Non-Target Impact of *P. euphyllurae*

#### 3.3.1. No Choice Tests

Although efforts were made to have nymphs of mixed ages on material of each host plant, it was not always possible to determine the exact numbers and stages of the wax-covered psyllids before the tests because lifting or removing the wax could lead to the permanent displacement of the hosts. Unfortunately, it was later discovered that wax is not a good indicator of psyllid presence. Overall, no psyllids (dead or alive) were found in 21% of the cages at the end of the experiment (Table 3). This percentage was the highest for *E.* nr. *robusta* cages (68%) suggesting that it is either a highly mobile and/or easily disturbed psyllid species. In contrast, psyllids or psyllid exoskeletons were found in all *E. olivina* (T) cages and in 82% of the *N. arctostaphyli* cages (Table 3). Despite this drawback, seven *P. euphyllurae* adult wasps were reared from those tests, and all individuals emerged from the target host *E. olivina* (Table 3). 

#### 3.3.2. Choice Tests 

A total of 62 observations (replicates) were conducted but only 37 wasps were responsive and demonstrated a clear searching behavior. Upon their release, those responsive females usually started walking in a random pattern while antennating the surface of the petri dish until they encountered host plant parts (= first choice). Then, the females would or would not climb on the plant parts to continue this searching behavior. The target host plant was the first choice in 69%–73% of the observations (Table 4). 

Host patch time was significantly longer on the target host plants than the non-target host plants, at least for two of the three non-target species tested: *E.* nr. *robusta* (Table 4; t = 2.69, df = 6.7, *p* = 0.032) and *C. nigrella* (t = 4.57, df = 6, *p* = 0.004). There was no significant difference in patch residence time when searching on manzanita vs. olive plant parts (Table 4; t = 0.30, df = 8.4, *p* = 0.770).

Encounters with the target host plant always led the wasps to start searching the plant parts, and encounters with wax always triggered probing behavior (Table 5). Searching and probing led to host finding in 53%–80% of the cases, and parasitoid attack always followed host finding. However, attacks did not always end in oviposition because oviposition attempts sometimes caused the psyllid host to flee and successfully escape the attack. 

Probing on non-target plant species was observed in one test only with manzanita (Table 5). However, it did not lead to host finding, and, eventually, the wasp left the non-target plant species to investigate the target host plant where it searched and probed the surface. However, that wasp was unsuccessful at locating a host because of the unusually thick layer of wax protecting them in this specific replicate (most wax varies in thickness from 2–10 mm). In the remaining observations on non-target plant species, probing was never observed (Table 5). 

Oviposition was different from probing in terms of duration and wasp movement. While probing was characterized by quick insertions (less than second) of the ovipositor into the substrate (wax or host), oviposition lasted longer (2.21 ± 0.20 (SE) min, n = 16). Additionally, the wasp remained completely motionless during oviposition, a clear contrast with the restless activity during searching and probing. Oviposition attempts often resulted in the host fleeing the attack. However, once the ovipositor was inserted into the host, they seemed temporarily paralyzed for the duration of oviposition but were usually able to walk away soon after the attack. 

Host feeding behavior was never observed for young (1–12 d) inexperienced or older (16–24 d) experienced host-deprived females. During the additional observations with the older females, all of them were able to successfully find a host after searching and probing the target host plant, while three of them were able to successfully attack and oviposit in a host.

About 40% of the parasitoid females tested did not respond to either target or non-target psyllids, spending most of their time motionless or grooming on the side or floor of the petri dish. In our observations, non-responsive *P. euphyllurae* females tended to be younger (3.3 ± 0.7 d, n = 25) than responsive females (6.4 ± 0.8, n = 37; t = 2.67, df = 59.9, *p* = 0.01). 

## 4. Discussion

First detected in southern California (Orange County), *E. olivina* has expanded its distribution northward to Carmel Valley on the central coast of California, 566 km northward, or about 51 km per year. Current distribution appears to be limited to coastal areas of California between Monterey and San Diego; there are no reports of infestations on olives in the major production areas of central and northern California. Sampling in 2009, 2014, and 2015 in southern California found no primary parasitoid attacking the olive psyllid, only generalist predators that are unlikely to exert the suppression required to control populations of olive psyllids [15], confirming the need for introducing more specialized (co-evolved) natural enemies. 

Results from our foreign collections supported previous studies on the host preference of the candidate parasitoid, *P. euphyllurae*, which has been reported only from the olive psyllid infesting olive trees in the western Mediterranean Basin [4,5,6]. In our collections, it was the major primary parasitoid emerging from olive psyllid nymphs in Spain. In 2014 and 2015, a low proportion of *P. pulchellus* was recovered. This was the first time that this species has been described developing on *E. olivina* [16]. However, the low reproduction rate obtained on *E. olivina* in a follow-up laboratory experiment suggested that the olive psyllid may not be its preferred host [16]. Indeed, we did not recover any *P. pulchellus* in 2013 and 2018. 

A parasitoid host range assessment is often the first step in the development of biological control programs using parasitoids as control agents [17]. No *P. euphyllurae* adults emerged from the two tested non-target species in the no-choice tests, whereas the parasitoid emerged from the exposed target hosts. These results were further confirmed through direct observations during the choice tests. None of the three tested non-target species were attacked or parasitized by *P. euphyllurae*. Most importantly, no parasitism occurred on *N. arctostaphyli*, the most closely related psyllid to the target psyllid, suggesting that attack and reproduction on more distantly related psyllids native to California are even less likely. These results are supported by a previous study assessing the host specificity of *P. euphyllura* [12]. This study found that no reproduction occurred on six tested non-targets exposed to this parasitoid during no-choice sequential tests and choice tests. 

Behavioral observations also revealed that, while both *E.* nr. *robusta* and *C. nigrella* did not trigger any interest by the parasitoid, the manzanita cuttings seemed to retain the parasitoid a little longer. Honeydew is a known volatile and contact kairomone acting as an attractant and host searching stimulant for several parasitoid species [18,19,20], including psyllid parasitoids [21]. As the sugar composition of honeydew is determined by the insect species [22], and since *E. olivina* and *N. arctostaphyli* are closely related species, it is likely that they have a similar honeydew composition signature, inducing longer patch residence time in *P. euphyllurae*. However, only one single probing attempt on manzanita was observed in our study, suggesting that *N. arctostaphyli* failed to provide the necessary additional chemical or physical cues resulting in host location and acceptance. 

Although host feeding seems to be a common behavior in *Psyllaephagus* species [9,21], usually resulting in increased host mortality, this behavior was not recorded in our observations with both young and older host-deprived females. However, our observations did highlight another possible cause of non-reproductive mortality by *P. euphyllurae*. As the parasitoids narrowed their search in the immediate vicinity of a psyllid and the probing intensified, the wasps sometimes inadvertently probed the host through the wax cover, resulting in the sudden disturbance and relocation of the psyllid as it attempted to escape parasitism. Such disturbed psyllids were observed walking around the small petri dish for the rest of the observation, never settling back down on the plant. In natural settings, this wandering may lead to death, especially if the psyllid has been mutilated by the probing and/or if it drops to the ground where it is exposed to desiccation, starvation, and/or predation. Abram et al. [23] recommended this non-reproductive mortality be assessed when evaluating for non-target impact. As no probing was observed on non-target species in our study, it is unlikely that *P. euphyllurae* will negatively affect them in this manner. 

In many parasitoid species (usually synovigenic species), newly emerged females require additional time (several days) to mature their eggs (preoviposition period). In our study, non-responsive *P. euphyllurae* females tended to be younger, and may have needed more time to mature their eggs, although there are numerous other genetic, physiological, and/or environmental factors that could affect a parasitoid response to the presence of hosts [24].

## 5. Conclusions

Our study showed that *E. olivina* is slowly spreading through the state with the potential to reach the olive production region of California’s Central Valley. The surveys also confirmed the absence of local specific natural enemies capable of controlling this psyllid. Therefore, the permanent establishment of a co-evolved parasitoid specializing on the olive psyllid is the next option to manage this pest and slow its spread. Foreign collections confirmed that *P. euphyllurae* is the main primary parasitoid in the region of origin of the olive psyllid and may be a good candidate for release. Our initial evaluation of this parasitoid’s potential non-target impact showed great host specificity to the olive psyllid. However, our host range assessment was limited to three native North American psyllid species and may need to be expanded to include more non-target species. Habitat complexity and chemical inputs in agriculture can create different conditions than occur in controlled laboratory settings. However, while our lab study lacks the complexity of the real world in which these insects interact, it is by far the most conservative setting to conduct such safety tests. If a parasitoid does not attack the presented host in this setting, it will be very unlikely to ever happen in an agricultural landscape.

## Figures and Tables

**Table 1:** Locations of the surveyed sites in Spain.

Waypoint	Latitude	Longitude	Nearest City, Province
46	N40.66213°	E000.58365°	Amposta, Catalonia
281	N40.57948°	E000.55220°	Amposta, Catalonia
380	N40.54241°	E000.31494°	Sant Mateu, Valencia
274	N38.39974°	W001.38686°	Jumillo, Murcia
375	N37.56890°	W001.47208°	Murcia (coast), Murcia

**Table 2:** Numbers (relative abundance) of primary and secondary parasitoids emerging from mummified olive psyllids collected in Spain in 2013–2018.

Year	Province	*P. Euphyllurae*	*P. Pulchellus*	*A. Trapezoidea*	*Pachyneuron* sp.
2013	Catalonia	136	0	101	1
	Valencia	8	0	0	0
	Murcia	83	0	0	0
	total	227 (69%)	0 (0%)	101 (31%)	1 (<1%)
2014	Catalonia	89	8	223	73
	Valencia	65	5	15	1
	Murcia	94	28	5	16
	total	248 (40%)	41 (7%)	243 (39%)	90 (14%)
2015	Catalonia	226	69	240	40
	Murcia	108	30	4	8
	Valencia	25	16	0	5
	total	359 (46%)	115 (15%)	244 (32%)	53 (7%)
2018	Catalonia	79	0	109	15
	Valencia	149	0	79	12
	Murcia	198	0	1	99
	total	426 (57%)	0 (0%)	189 (26%)	126 (17%)
	Grand total	1260 (51%)	156 (6%)	777 (32%)	270 (11%)

**Table 3:** Non-target species tested in the sequential no-choice tests with numbers of replicates (n) for each sequence of exposure, and numbers of adult *Psyllaephagus euphyllurae* reared from each target (T) and (NT) hosts. It was later discovered that wax is not a good indicator of psyllid presence and that we had set up a number of cages with no psyllid hosts.

NT Species Tested	Sequence	n	Host Plant	*P. Euphyllurae* Reared	No. of Cages with No Psyllids
*E.* nr. *robusta* (deerbrush)	NT - T	20	NT	0	15 (75%)
			T	2	0
	T - NT	18	NT	0	11 (61%)
			T	2	0
*N. arctostaphyli* El Dorado Co. (manzanita)	NT - T	15	NT	0	0
			T	0	0
	T - NT	8	NT	0	2 (25%)
			T	1	0
*N. arctostaphyli* Napa Co. (manzanita)	NT - T	6	NT	0	2 (33%)
			T	2	0
	T - NT	10	NT	0	3 (30%)
			T	0	0

**Table 4:** Total numbers of observations (n) for each choice test, numbers of observations where target or non-target host plants was first encountered (first choice), and mean host patch time in min (±SE) on target and non-target host plants.

NT Species Tested	n	Host Plant	First Choice	Patch Time
*N. arctostaphyli* vs. *E. olivina*	16	T	11	15.9 ± 3.54
(manzanita vs. olive)		NT	5	14.1 ± 4.88
*E.* nr. *robusta* vs. *E. olivina*	11	T	8	16.6 ± 3.50
(deerbrush vs. olive)		NT	3	3.7 ± 3.26
*C. nigrella* vs. *E. olivina*	10	T	7	20.1 ± 4.38
(skunkbush sumac vs. olive)		NT	3	0.1 ± 0.01

**Table 5:** Total number of observations (n) on target and non-target host plants with numbers of observations where female parasitoids were seen probing the substrate (Pr), host finding (Hf.), attacking (At.), and ovipositing (Ov.) in a psyllid host.

Host Plant (Target vs. Non-Target)	On Target Host				On Non-Target Host				
	n	Hf.	At.	Ov.	n	Pr.	Hf.	At.	Ov.
Olive vs. manzanita	15	8	8	3	5	1	0	0	0
Olive vs. deerbrush	10	8	8	6	3	0	0	0	0
Olive vs. skunkbush sumac	10	8	8	7	3	0	0	0	0

## References

[1] Gill R., Watson G. (2007). *Euphyllura olivina* Costa–Psylloidea: Psyllidae. Olive tree psyllid. Calif. Plant Pest Dis. Rep..

[2] Percy D.M., Rung A., Hoddle M.S. (2012). An annotated checklist of the psyllids of California (Hemiptera: Psylloidea). Zootaxa.

[3] Jardak T., Smiri H., Moalla M., Khalfallah H., Cavallaro R., Crovetti A., Cavalloro R., Crovetti A. (1985). Tests to assess the damage caused by the olive psyllid *Euphyllura olivina* Costa (Homoptera, Psyllidae): Preliminary data on the harmfulness threshold. Integrated Pest Control in Olive Groves. Proceedings of the CEC/FAO/IBOC International Joint Meeting.

[4] Aversenq S., Gratraud C., Pinatel C. (2005). Ravageurs et auxiliaires des oliviers: Synthèse de trois ans d’observations dans le sud-est de la France. Phytoma.

[5] Mercet R.G. (1921). Fauna Ibérica. Himenópteros Fam. Encírtidos.

[6] Triapitsyn S.V., Jones J.M.L., Pickett C.H., Buffington M.L., Rugman-Jones P.F., Daane K.M. (2014). Description of the male of *Psyllaephagus euphyllurae* (Masi) (Hymenoptera, Encyrtidae), a parasitoid of the olive psylla, *Euphyllura olivina* (Costa) (Hemiptera, Liviidae), with notes on its reproductive traits and hyperparasitoids. J. Entomol. Acarol. Res..

[7] Noyes J.S. Universal Chalcidoidea Database. http://www.nhm.ac.uk/chalcidoids.

[8] Chauzat M.P., Purvis G., Dunne R. (2002). Release and establishment of a biological control agent, *Psyllaephagus pilosus* for eucalyptus psyllid (*Ctenarytaina eucalypti*) in Ireland. Ann. Appl. Biol..

[9] Daane K.M., Sime K.R., Dahlsten D.L., Andrews J.W., Zuparko R.L. (2005). The biology of *Psyllaephagus bliteus* Riek (Hymenoptera: Encyrtidae), a parasitoid of the red gum lerp psyllid (Hemiptera: Psylloidea). Biol. Control.

[10] Dahlsten D., Hansen E., Zuparko R., Norgaard R. (1998). Biological control of the blue gum psyllid proves economically beneficial. Calif. Agric..

[11] Chermiti B., Hawlitzky N., Boulay C., Onillon J. (1986). Some development characteristics in the endoparasite insect *Psyllaephagus euphyllurae* (Hymenoptera: Encyrtidae) and feeding on its host, *Euphyllura olivina* (Homoptera: Psyllidae). Entomophaga.

[12] Pickett C.H. (2019). Personal Communication.

[13] Del Bene G., Gargani E., Landi S. (1997). Observations on the life cycle and diapause of *Euphyllura olivina* (Costa) and *Euphyllura phillyreae* Foerster (Homoptera Aphalaridae). Adv. Hortic. Sci..

[14] Van Alphen J.J.M., Jervis M.A., Jervis M.A., Kidd N. (1996). Foraging behavior. Insect Natural Enemies: Practical Approaches to Their Study and Evaluation.

[15] Johnson M.W., Daane K.M., Lynn-Patterson K. (2010). Appraising the threat of olive psyllid to California table olives. Final & Interim Research Reports.

[16] Jones J.M., Pickett C.H., Triapitsyn S.V., Hoddle M.S. (2016). New host record for *Psyllaephagus pulchellus* (Mercet, 1921) (Hymenoptera, Encyrtidae) as a parasitoid of *Euphyllura olivina* (Costa, 1839) (Hemiptera, Liviidae), in Spain. Boln. Asoc. Esp. Ent..

[17] Van Driesche R.G., Reardon R. (2004). Assessing Host Ranges for Parasitoids and Predators Used for Classical Biological Control: A Guide to Best Practice.

[18] Bouchard Y., Cloutier C. (1984). Honeydew as a source of host-searching kairomones for the aphid parasitoid *Aphidius nigripes* (Hymenoptera: Aphidiidae). Can. J. Zool..

[19] Budenberg W. (1990). Honeydew as a contact kairomone for aphid parasitoids. Entomol. Exp. Appl..

[20] Vinson S., Harlan D., Hart W. (1978). Response of the parasitoid *Microterys flavus* to the brown soft scale and its honeydew. Environ. Entomol..

[21] Mehrnejad M.R., Copland M.J. (2006). Behavioral responses of the parasitoid *Psyllaephagus pistaciae* (Hymenoptera: Encyrtidae) to host plant volatiles and honeydew. Entomol. Sci..

[22] Hendrix D.L., Wei Y.-A., Leggett J.E. (1992). Homopteran honeydew sugar composition is determined by both the insect and plant species. Comp. Biochem. Phys. B.

[23] Abram P.K., Brodeur J., Urbaneja A., Tena A. (2019). Nonreproductive effects of insect parasitoids on their hosts. Annu. Rev. Entomol..

[24] Lewis W., Vet L.E., Tumlinson J., Van Lenteren J., Papaj D. (1990). Variations in parasitoid foraging behavior: Essential element of a sound biological control theory. Environ. Entomol..

